# Action of Dallas County Medical Society on the International Congress Question

**Published:** 1885-08

**Authors:** Goodwin E. Peters

**Affiliations:** Sect’y.; Dallas, Tex.


### SOCIETY NOTES.

### Action of the Dallas County Medical Society on the International Congress Question.

THE following has been sent us with the request to publish it. We do so as a duty we, as a Journalist, owe the profession. We cannot say "it affords us pleasure," for it does not. Indeed it pains and mortifies us, very much, to discover that there is a division on this subject, where we believed the utmost unanimity of sentiment prevailed.

DALLAS, TEX., July 29, 1885.

F. E. DANIEL, M. D., AUSTIN, TEXAS.

*Dear Sir:*—Will you kindly insert the following preamble and resolutions passed by the Dallas County Medical Society, on July 25th, in the columns of your Journal:

WHEREAS, The American Medical Association, at its meeting in Washington, D. C., May, 1884, recognized a general desire of the medical profession of the United States, by adopting a resolution under which a committee was appointed, whose duty it should be to extend an invitation to the International Medical Congress, shortly to assemble at Copenhagen, to hold its next meeting in 1887 at Washington, D. C.; and

WHEREAS, The said committee, by the letter and spirit of this resolution, was fully empowered to act, not only as a committee of invitation, but as an executive committee as well; and

WHEREAS, The said committee, in pursuance of the objects of the above mentioned resolution, and duly exercising the unlimited authority delegated to it, enlarged its membership, and otherwise provided for the successful holding of an International Medical Congress at Washington in 1887,all of which arrangements were considered by us as judicious, and contrary to what has been charged by some, wholly disinterested as to personal or local aggrandizment; and

WHEREAS, The American Medical Association at its last meeting at New Orleans,did, in our judgement,unwisely and untimely, virtually rescind its former action, which reactionary movement has deranged, if not indefinitely suspended, the work of the original committee, which was satisfactorily progressing, and created an indifference to the Congress among the recognized leaders of medical thought and interest throughout the country; and

WHEREAS, There are those who persist in urging the so-called justice of their claims for the organization of the International Medical Congress on a territorial basis, which unfortunate idea has been unwisely further extended by some members of the profession in Texas, in a manner calculated to arouse a sectional prejudice which has little, if any, existence in our State; therefore be it

*Resolved*, That the Dallas County Medical Society deplores what must be considered the present inter-regnum in the affairs of the contemplated International Medical Congress, brought about, as we believe, by an ill-considered, hasty action of the American Medical Association at the New Orleans meeting before mentioned; that this Society was fully satisfied with the work of the original committee which was composed of able, eminent and conscientious members of the profession ; that this Society repudiates any attempt to inject a sectional feeling into a purely professional matter only, and that said attempt, if offered in behalf of the medical profession of Texas, is, in the opinion of this Society, both unauthorized and gratuitous; and that looking beyond a narrow-minded policy of personal aggrandizement and sectional interest, we heartily commend the recent action of Philadelphia and New York brethren, as well as those elsewhere, who have retired from the Congress until a more dignified and unselfish view of arrangements can be had; and we pledge them our hearty support and good will, in their efforts to advance the interests of the American Medical Profession in future meetings of the International Medical Congress, where the truly representative abilities of our country shall be enlisted, uncontrolled by geographical lines, or personal preference.

GOODWIN E. PETERS, M. D., Sect'y.

